# Preface

**DOI:** 10.1155/S0962935194000669

**Published:** 1994

**Authors:** P. L. Giorgi, N. Oggiano, A. Kantar, A. Corrias

**Affiliations:** 1 Department of Paediatrics University of Ancona Italy; 2 Chair of the Paediatric Bronchopneumology Subgroup of the Italian Paediatric Society Italy


					Preface
Mediators of Inflammation 3, S3 (1994)
Preface
There is, nowadays, enough evidence for the pres-
ence of an inflammatory process in the airways of all
asthmatics. Airways inflammation is a multifactori-
ally-mediated process involving various cells along
with humorally and tissue-derived mediators. Our
therapeutic armamentarium for inflammatory dis-
eases of the airways is currently limited. Although
effective and safe treatment of asthma is no longer an
elusive target, the widespread requirement for drugs
that offer greater efficacy and fewer complications
than steroids has shifted our research interest to-
wards an improved understanding of the mecha-
nisms involved in airway inflammation.
Since their introduction in Italy, sodium
cromoglycate (1971) and nedocromil sodium (1989)
have generated a large database of information in
many scientific areas. The aim of this workshop
is to bring together Italian experts in paediatric
bronchopneumology to describe their experiences
and highlight advances achieved in the clinical and
biochemical knowledge of these drugs.
It is the hope of the organizers that this workshop
will meet the scientific and medical need for a review
of the 'Italian experience' of the use of these non-
steroidal anti-inflammatory drugs in asthma.
P. L. Giorgi
N. Oggiano
A. Kantar
A. Corrias2
1Department of Paediatrics,
University of Ancona, Italy and
2Chair of the Paediatric Bronchopneumology
Subgroup of the Italian Paediatric Society
() 1994 Rapid Communications of Oxford Ltd Mediators of Inflammation. Vol 3 (Supplement). 1994 $3

				
